# Relationship between Ocular Deviation and Visual Function in Retinitis Pigmentosa

**DOI:** 10.1038/s41598-018-33211-6

**Published:** 2018-10-05

**Authors:** Manabu Miyata, Akio Oishi, Ken Ogino, Maho Oishi, Tomoko Hasegawa, Yuko Nagasaku, Hanako Ohashi Ikeda, Hiroshi Ohtsuki, Akitaka Tsujikawa

**Affiliations:** 1 Department of Ophthalmology and Visual Sciences, Kyoto University Graduate School of Medicine, Kyoto, Japan; 2 Department of Ophthalmology, Okayama University Graduate School of Medicine, Dentistry, and Pharmaceutical Sciences, Okayama, Japan

## Abstract

In retinitis pigmentosa (RP), peripheral visual-field loss starts in early stages, whereas central vision loss occurs in advanced stages. Sensory strabismus gradually occurs in RP. We investigated the relationship between ocular deviation and visual function and explored for sensory strabismus risk factors in 119 consecutive patients with RP at various stages. We assessed ocular deviation at far and near distances, that is the central visual field, using the mean deviation (MD) value and visual acuity (VA), and the residual binocular field area, using Goldmann perimetry (GP), in 33 patients. The horizontal ocular deviation at near distance was >10° in 30% patients and correlated with residual visual function. Although there was no effective cut-off value for central visual function, a cut-off residual GP area of 40 cm^2^ distinguished patients with a larger from those with a smaller horizontal ocular deviation at far distance (*P* = 0.04). Our findings suggest that visual function is negatively associated with ocular deviation in patients with RP and that the sensory strabismus risk is relatively high for patients with a binocular visual field <40 cm^2^. Thus, screening for ocular alignment may be necessary for patients with RP-associated severe vision loss as part of their comprehensive care.

## Introduction

Retinitis pigmentosa (RP) causes progressive loss of visual function, including the visual field and visual acuity (VA), with a decrease in retinal blood flow^1^ due to rod and subsequent cone photoreceptor cell death^2^. As a result, peripheral visual field loss starts in the early stage, whereas central vision loss occurs in advanced stages. This is partly shown by recent studies using optical coherence tomography (OCT) that have demonstrated a progressive ellipsoid zone band narrowing from the periphery to the centre as the disease progresses^3–6^.

Loss or impairment of vision induces sensory strabismus. The direction of horizontal strabismus depends on the age at the onset of vision loss. Late-onset vision loss induces exotropia more frequently than does congenital- or early-onset vision loss^7,8^. Patients with RP typically lose night vision in adolescence, peripheral vision in young adulthood, and central vision in later life^2^. Therefore, these patients are expected to exhibit sensory exotropia, although it remains unclear whether patients in more advanced stages of RP show greater deviation than those in early stages.

Peripheral fusion^9^ significantly contributes towards the temporary stabilisation of relative eye position^10^. Patients who lose peripheral fusion cannot stabilise their ocular alignment and consequently develop strabismus. However, the central visual field and VA may also affect the ocular position. Patients with RP show various degrees of concentric visual field defects due to preceding peripheral retinal degeneration^11,12^. In addition, a considerable area of the peripheral visual field occasionally remains intact, despite the small central visual field, forming the characteristic ring scotoma observed in RP, which enables separate assessments of the effects of peripheral and central visual fields on ocular deviation. This makes RP a unique model compared with other diseases that affect the visual field, such as glaucoma, which rarely cause central without peripheral vision loss. Thus, we consider RP to be a unique model for assessing the relationship between ocular deviation and visual function.

Accordingly, the aim of the present study was to investigate the relationship between ocular deviation and visual function and explore the risk factors for sensory strabismus in patients with RP at various stages.

## Results

This study included 119 consecutive patients with RP (Table 1), including two patients with no light perception who were each assigned a minimum mean deviation (MD) value of −34.74 dB (a 55-year-old patient) and −33.32 dB (an 83-year-old patient), respectively. The mean patient age, logarithm of the minimal angle of resolution (logMAR), MD value, and residual Goldmann perimetry (GP) area were 48.4 ± 19.6 years, 0.25 ± 0.45, −17.2 ± 10.6 dB, and 59.7 ± 58.5 cm^2^, respectively. The horizontal deviation at far distance (HDF), horizontal deviation at near distance (HDN), vertical deviation at far distance (VDF), and vertical deviation at near distance (VDN) were 3.7° ± 5.2°, 7.8° ± 7.3°, 0.4° ± 1.4°, and 0.4° ± 1.0°, respectively. Phoria, phoria–tropia, and tropia were observed in 81 (68%), 8 (7%), and 30 (25%) patients, respectively. Exodeviation, orthophoria, and esodeviation at near distance were observed in 98 (82%), 8 (7%), and 13 (11%) patients, while those at far distance were observed in 82 (79%), 15 (14%), and 7 (7%) patients, respectively. VDN and VDF were observed in 23 (19%) and 22 (21%) patients, respectively. The age of patients with exodeviation (49.5 ± 19.5 years) was significantly greater than that of patients with esodeviation at near distance (37.5 ± 18.2 years, *P* = 0.04), with no significant difference from the age of patients with esodeviation at far distance (*P* = 0.74). Whereas 15 patients (13%) required the Krimsky test, the remaining underwent the prism and alternate cover test (PACT) at near and far distances.

**Table 1:** Characteristics of patients with retinitis pigmentosa and comparison between the large- and small-angle groups.

		Total	Large-Angle Group	Small-Angle Group	*P*
Number of patients		119	36	83	
Age, years (range)		48.4 ± 19.6 (8–90)	53.4 ± 19.5 (10–83)	46.3 ± 19.3 (8–90)	0.07
Sex (M/F)		58/61	18/18	40/43	0.86^#^
Axial length (N = 86), mm		24.2 ± 1.6	24.1 ± 1.6^a^	24.2 ± 1.7^b^	0.73
LogMAR		0.25 ± 0.45	0.39 ± 0.53	0.18 ± 0.39	0.04*
MD value, dB		−17.2 ± 10.6	−19.5 ± 10.0	−16.1 ± 10.7	0.11
Interocular difference	LogMAR	0.23 ± 0.48	0.41 ± 0.72	0.15 ± 0.31	0.04*
	MD value, dB	2.1 ± 2.9	2.8 ± 3.4	1.8 ± 2.6	0.15
Residual GP area (N = 33), cm^2^		59.7 ± 58.5	32.1 ± 52.5	68.6 ± 58.6	0.13
Deviation at near distance (N = 119), degrees	Horizontal	7.8 ± 7.3	16.3 ± 7.3	4.1 ± 2.7	<0.001*
	Vertical	0.4 ± 1.0	0.6 ± 1.0	0.3 ± 0.9	0.11
Deviation at far distance (N = 104), degrees	Horizontal	3.7 ± 5.2	8.6 ± 7.2^c^	1.9 ± 2.5^d^	<0.001*
	Vertical	0.4 ± 1.4	0.6 ± 1.0^e^	0.4 ± 1.5^f^	0.57
Phoria/phoria–tropia/tropia		81/8/30	9/7/20	72/1/10	<0.001*^##^

Data are presented as means ± standard deviations where applicable.

Large-angle group: patients with a horizontal ocular alignment of ≥10° at near distance.

Small-angle group: patients with a horizontal ocular alignment of <10° at near distance.

Residual GP area: total residual visual field area for the right and left eyes, as determined by Goldmann perimetry.

logMAR, logarithm of the minimal angle of resolution; MD value, mean deviation value obtained using the 10-2 Swedish Interactive Threshold Algorithm standard program of the Humphrey field analyser.

Axial length and logMAR data are presented as average values for the right and left eyes.

Data in ^a,b,c,d,e^and ^f^are missing for 5, 28, 8, 7, 8, and 7 patients, respectively.

Ocular deviation was been analysed using absolute values, regardless of the strabismus type.

^#^Chi-square test; ^##^Chi-square trend test; remaining, *t*-tests.

*S*t*atistically significant (*P* < 0.05).

**Figure 1:**
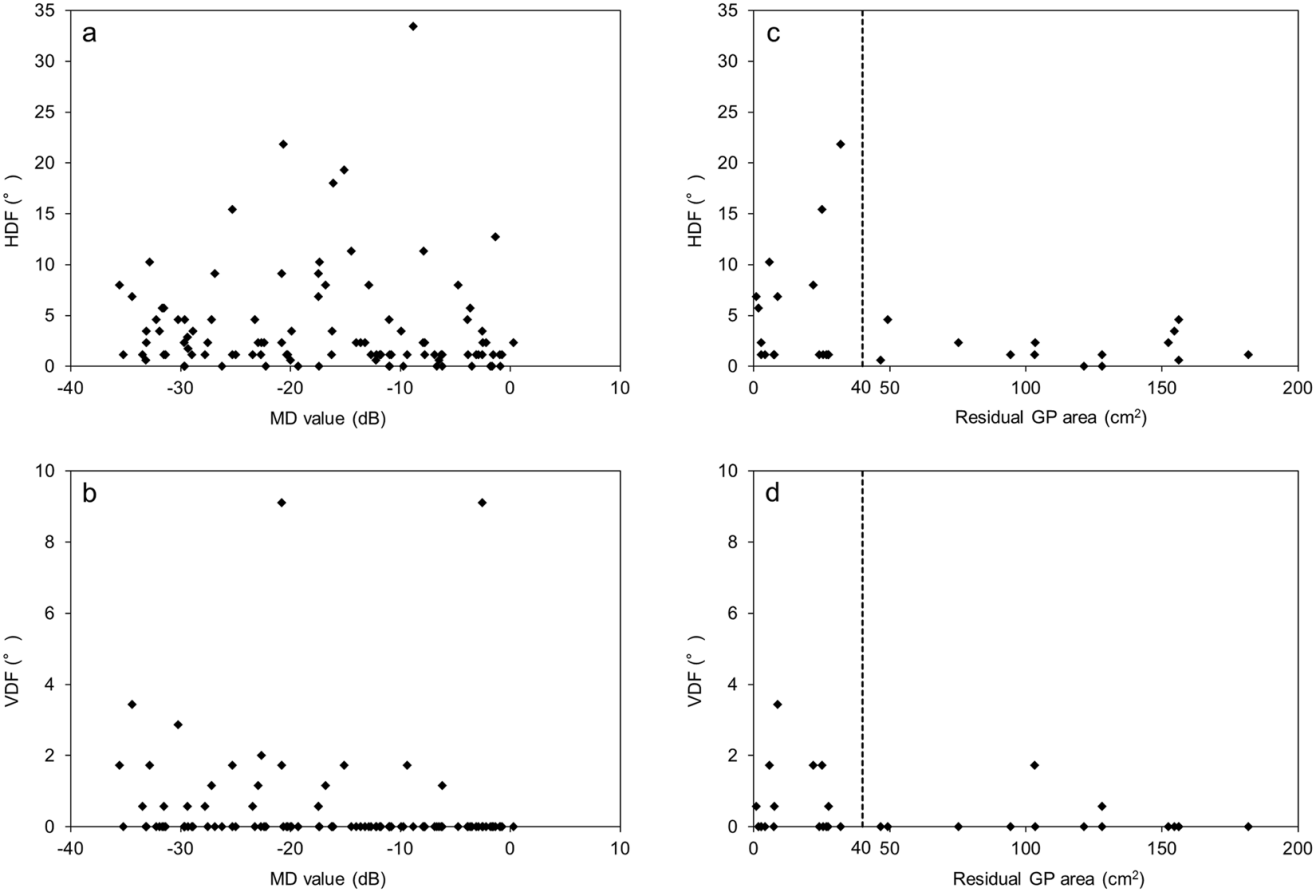
Relationship between ocular deviation at far distance and the visual field in patients with retinitis pigmentosa. (**a**) The horizontal deviation at far distance (HDF) negatively correlated with the mean deviation (MD) value (*P* = 0.04; r = −0.20). (**b**) The vertical deviation at far distance (VDF) also negatively correlated with the MD value (*P* = 0.004, r = −0.28). There were no effective cut-off MD values for distinguishing patients with a larger ocular deviation from those with a smaller ocular deviation. (**c**,**d**) Both HDF and VDF tend to exhibit a correlation with the residual Goldmann perimetry (GP) area (*P* = 0.08 and 0.13, respectively). A cut-off residual GP area of 40 cm^2^ can be set to distinguish patients with a larger deviation from those with a smaller deviation.

Horizontal deviation values showed a significant or marginally significant correlation with the patient age, VA, MD value, interocular difference in VA, residual GP area, and binocularity status. While VDF exhibited a similar trend, VDN did not show any correlation with the investigated factors (Table 2). Figure 1 shows the correlation of ocular deviation at far distance with the MD value and residual GP area. There was no effective cut-off value for distinguishing patients with a larger ocular deviation from those with a smaller one. However, a cut-off residual GP area of 40 cm^2^, corresponding to a central circular area of approximately 30°, could distinguish patients with a larger ocular deviation from those with a smaller ocular deviation (HDF, 5.1° ± 6.0° vs 1.8° ± 1.5°, *P* = 0.04; VDF, 0.6° ± 1.0° vs 0.2° ± 0.5°, *P* = 0.11, respectively). The large-angle group (near distance) exhibited a significantly worse VA and larger interocular difference in VA compared with the small-angle group (*P* *=* 0.04 for both), which exhibited a worse MD value and residual GP area compared with the large-angle group, although the differences were not statistically significant (Table 1).

**Table 2:** Correlation between horizontal and vertical ocular deviation at each distance and other parameters.

Parameter		HDN (N = 119)		HDF (N = 104)		VDN (N = 119)		VDF (N = 104)	
		*P*	r	*P*	r	*P*	r	*P*	r
Age		0.006*	0.25	0.04*	0.20	0.95	—	0.20	—
Sex		0.91	—	0.28	—	0.22	—	0.93	—
Axial length		0.81^a^	—	0.27^c^	—	0.81^a^	—	0.35^c^	—
LogMAR		0.02*	0.22	0.01*	0.25	0.88	—	0.07	0.18
MD value		0.12	—	0.04*	−0.20	0.89	—	0.004*	−0.28
Interocular difference	LogMAR	0.08	0.16	0.01*	0.24	0.94	—	0.13	—
	MD value	0.22	—	0.79	—	0.33	—	0.52	—
Residual GP area (N = 33)		0.03*	−0.39	0.08^d^	−0.32	0.83	—	0.13^d^	—
VDN/HDN		0.03*	0.27	<0.001*	0.42	0.03*	0.27	0.02*	0.23
VDF/HDF		0.02*^b^	0.23	0.002*	0.30	<0.001*^b^	0.42	0.002*	0.30
Phoria/phoria–tropia/tropia		<0.001*	−0.56	0.001*	0.30	<0.001*	−0.54	0.001*	0.32

Residual GP area: total residual visual field area for the right and left eyes, as determined by Goldmann perimetry.

HDN, horizontal deviation at near distance; HDF, horizontal deviation at far distance; VDN, vertical deviation at near distance; VDF, vertical deviation at far distance; logMAR, logarithm of the minimal angle of resolution; MD value, mean deviation value obtained using the 10-2 Swedish Interactive Threshold Algorithm standard program of the Humphrey field analyser.

Axial length and logMAR data are presented as average values for the right and left eyes.

Data in ^a,b,c^and ^d^are missing for 33, 15, 30, and 2 patients, respectively.

Ocular deviation was analysed using absolute values, regardless of the strabismus type.

*Statistically significant according to Spearman’s rank correlation coefficients (*P* < 0.05).

### Subanalysis for investigating wide visual field and ocular deviation association

In our subanalysis for investigating the association between wide visual field and ocular deviation, we included 30 patients after excluding two patients who could not undergo PACT at far distance because of a poor VA and one patient with interocular difference in logMAR ≥ 0.5 (Table 3). HDN correlated with VA, residual GP area, and binocularity status (*P* = 0.001, r = 0.56; *P* = 0.04, r = −0.37; *P* = 0.001, r = 0.56, respectively), but not with interocular differences in VA and MD value (Table 4). HDF, VDN, and VDF did not correlate with residual GP area (*P* = 0.13, 0.38, and 0.20, respectively). The results of this subanalysis were similar to those obtained with the overall analyses.

**Table 3:** Characteristics of patients with retinitis pigmentosa included in the subanalysis and comparison between the large- and small-angle groups.

		Total	Large-angle hroup	Small-angle group	*P*
Number of patients		30	7	23	
Age, years (range)		42.4 ± 18.7 (10–75)	45.3 ± 23.8 (10–75)	41.5 ± 17.4 (11–75)	0.65
Sex (M/F)		18/12	3/4	15/8	0.39^#^
Axial length (n = 19), mm		23.6 ± 1.6	23.1 ± 1.1	23.8 ± 1.7	0.38
LogMAR		0.13 ± 0.28	0.34 ± 0.32	0.06 ± 0.24	0.02*
MD value, dB		−16.2 ± 10.3	−19.8 ± 8.9	−15.1 ± 10.6	0.30
Interocular difference	LogMAR	0.10 ± 0.12	0.16 ± 0.14	0.08 ± 0.11	0.11
	MD value, dB	1.4 ± 1.8	2.2 ± 2.0	1.2 ± 1.8	0.20
Residual GP area, cm^2^		63.3 ± 59.5	35.8 ± 55.5	71.7 ± 59.3	0.17
Deviation at near distance, degrees	Horizontal	6.5 ± 6.1	14.6 ± 7.6	4.1 ± 2.6	0.01*
	Vertical	0.3 ± 0.8	0.8 ± 1.4	0.2 ± 0.5	0.29
Deviation at far distance, degrees	Horizontal	3.4 ± 4.7	7.4 ± 6.6	2.2 ± 3.3	0.009*
	Vertical	0.4 ± 0.8	0.6 ± 1.3	0.3 ± 0.6	0.43
Phoria/phoria–tropia/tropia		22/3/5	2/2/3	20/1/2	0.003*^##^

Data are presented as means ± standard deviations where applicable.

Large-angle group: patients with a horizontal ocular alignment of ≥10° at near distance.

Small-angle group: patients with a horizontal ocular alignment of <10° at near distance.

Residual GP area: total residual visual field area for the right and left eyes, as determined by Goldmann perimetry.

logMAR, logarithm of the minimal angle of resolution; MD value, mean deviation value obtained using the 10-2 Swedish Interactive Threshold Algorithm standard program of the Humphrey field analyser.

Axial length and logMAR data are presented as average values for the right and left eyes.

Ocualr deviation was analysed using absolute values, regardless of the strabismus type.

^#^Chi-square test; ^##^Chi-square trend test; remaining, *t*-tests.

*S*t*atistically significant (*P* < 0.05).

**Table 4:** Correlation between horizontal and vertical ocular deviation at each distance and other parameters used in the subanalysis (N = 30).

Parameter		HDN		HDF		VDN		VDF	
		*P*	r	*P*	r	*P*	r	*P*	r
Age		0.19	—	0.76	—	0.67	—	0.55	—
Sex		0.74	—	0.42	—	0.27	—	0.11	—
Axial length (N = 19)		0.58	—	0.42	—	0.73	—	0.34	—
LogMAR		0.001*	0.56	0.002*	0.55	0.34	—	0.57	
MD value		0.35	—	0.80	—	0.84	—	0.74	
Interocular difference	LogMAR	0.11	0.16	0.37	—	0.77	—	0.17	—
	MD value	0.14	—	0.40	—	0.22	—	0.71	—
Residual GP area		0.04*	–0.37	0.13	–0.32	0.38	—	0.20	—
VDN/HDN		0.15	—	0.11	—	0.15	—	0.12	—
VDF/HDF		0.12	—	0.09	0.32	0.11	—	0.09	0.32
Phoria/phoria–tropia/tropia		0.001*	0.56	0.001*	0.59	0.78	—	0.35	—

Residual GP area: total residual visual field area for the right and left eyes, as determined by Goldmann perimetry.

HDN, horizontal deviation at near distance; HDF, horizontal deviation at far distance; VDN, vertical deviation at near distance; VDF, vertical deviation at far distance; logMAR, logarithm of the minimal angle of resolution; VA, visual acuity; MD value, mean deviation value obtained using the 10-2 Swedish Interactive Threshold Algorithm standard program of the Humphrey field analyser.

Axial length and logMAR data are presented as average values for the right and left eyes.

Ocular deviation was analysed using absolute values, regardless of the strabismus type.

*Statistically significant according to Spearman’s rank correlation coefficients (*P* < 0.05).

Regarding the binocularity cut-off, there were differences in logMAR (*P* < 0.001), residual GP area (*P* < 0.001), HDN (*P* = 0.03), and HDF (*P* = 0.04) between the phoria and tropia groups (Table 5).

**Table 5:** Comparison between the phoria and tropia groups.

		Total	Phoria group	Tropia group	*P*
Number of patients		30	23	7	
Age, years (range)		42.4 ± 18.7 (10–75)	39.6 ± 51.4 (10–75)	51.4 ± 18.1 (17–75)	0.15
Sex (M/F)		18/12	15/8	3/4	0.29^#^
Axial length (n = 19), mm		23.6 ± 1.6	23.6 ± 1.6	23.4 ± 1.7	0.85
LogMAR		0.13 ± 0.28	0.03 ± 0.19	0.45 ± 0.30	<0.001*
MD value, dB		−16.2 ± 10.3	−14.8 ± 10.2	−20.9 ± 9.6	0.17
Interocular difference	LogMAR	0.10 ± 0.12	0.07 ± 0.09	0.17 ± 0.17	0.20
	MD value, dB	1.4 ± 1.8	1.3 ± 1.9	1.8 ± 1.7	0.52
Residual GP area, cm^2^		63.3 ± 59.5	77.3 ± 60.8	17.3 ± 18.7	<0.001*
Deviation at near distance, degrees	Horizontal	6.5 ± 6.1	4.4 ± 3.1	13.6 ± 8.4	0.03*
	Vertical	0.3 ± 0.8	0.2 ± 0.5	0.8 ± 1.4	0.29
Deviation at far distance, degrees	Horizontal	3.4 ± 4.7	1.7 ± 1.7	8.9 ± 7.1	0.04*
	Vertical	0.4 ± 0.8	0.2 ± 0.5	0.8 ± 1.3	0.28

Data are presented as means ± standard deviations where applicable.

Phoria group: patients with phoria or phoriatropia.

Tropia group: patients with tropia.

Residual GP area: total residual visual field area for the right and left eyes, as determined by Goldmann perimetry.

logMAR, logarithm of the minimal angle of resolution; VA, visual acuity; MD value, mean deviation value obtained using the 10-2 Swedish Interactive Threshold Algorithm standard program of the Humphrey field analyser.

Axial length and logMAR data are presented as average values for the right and left eyes.

Ocular deviation was analysed using absolute values, regardless of the strabismus type.

^#^Chi-square test; remaining, *t*-tests.

*Statistically significant (*P* < 0.05).

## Discussion

The present study showed that visual function is negatively associated with ocular deviation in patients with RP. Considering the correlation coefficient, the peripheral visual field seems to be more important than the central visual field for the stabilisation of ocular alignment. A residual binocular GP area >40 cm^2^, corresponding to a central circular GP area of approximately 30°, may be a practical cut-off value for screening high-risk patients. Furthermore, VA correlated with MD (*P* < 0.001) and with the interocular difference in VA (*P* = 0.01), but not with the residual GP area (*P* = 0.62). Thus, the residual GP area and central visual function, including VA, MD, and the interocular difference in VA, separately affect ocular deviation in patients with RP.

Both the mean VA and interocular difference in VA showed weak correlations with HDF. In patients with a poor VA, photoreceptors in the macular lesion are probably damaged; therefore, a small interocular difference during disease progression would affect the interocular difference in VA. Indeed, VA correlated with the interocular difference in VA (*P* = 0.01). Furthermore, a decrease in visual function in one eye also induces strabismus, which is known as sensory heterotropia^7^. Thus, it is possibile that the interocular difference in VA causes sensory heterotropia.

In a previous study, VDN was observed in 13% of patients with RP (n = 23)^13^; this rate is similar to that observed in the present study (19%). However, the vertical deviation observed in the present study population was small (VDF, 0.4° ± 1.4° vs. HDF, 3.7° ± 5.2°). In patients with impaired vision, a small vertical deviation will neither cause any binocularity disorder nor lead to complaints about aesthetics. Therefore, vertical deviation may have low clinical relevance for most patients, with the exception of some patients with a large deviation.

In the present study, esodeviation at near and far distances was observed in 11% (13% without orthophoria) and 7% (9% without orthophoria) of patients, respectively. These findings are in agreement with those of a previous report on patients with acquired vision loss (10% without orthophoria)^8^. Consistent with the fact that RP induces acquired vision loss, the number of patients with sensory exotropia was higher than that of patients with esotropia in the present study^7,8^. Furthermore, patients with exodeviation were significantly older than those with esodeviation at near distance, but not those with esodeviation at far distance. A possible reason for this could be an increase in near exodeviation due to a decrease in convergence ability with age^14^.

The lifetime risk of being diagnosed with adult-onset strabismus is approximately 4%^15^. In the present study, 30% patients exhibited HDN >10°. This incidence rate is obviously higher than that for the general population. The present study showed not only a correlation between visual function and ocular deviation but also a high rate of strabismus with surgical indications in patients with RP. Strabismus negatively affects self-esteem in patients with a deviation ≥25 prism dioptres^16^. Hunter mentioned that strabismus surgery, even in patients with no potential for binocular vision, allows an individual to communicate normally with others^17^. In a previous study, all patients who underwent treatment for strabismus >20 prism dioptres were satisfied even though they could not see the outcome^18^. Thus, cosmetic strabismus surgery should be considered, for patients who desire the surgery, even those with advanced RP.

This study has some limitations. First was it had a cross-sectional design. It may be more convincing if changes in ocular deviation are demonstrated in accordance with changes in vision loss. Further longitudinal studies are necessary to overcome this limitation. Second, ocular deviation at far distance could not be measured in patients with a poor VA. Although ocular deviation at near distance is affected by convergence, which is unstable, and although the Krimsky test is inferior to PACT in terms of accuracy, patients in advanced stages of RP cannot gaze at a fixation target at a far distance. In this study, we also included patients who could not undergo the far distance test, because the exclusion of patients with common RP would bias the results. Third, we did not assess binocular visual function, as most patients exhibited ambiguous responses in binocular testing using Bagolini striate glasses during the preliminary examinations. Fourth, causative gene mutations were not taken into consideration. At present, approximately 70 genes have been identified to be associated with RP, and variations in these genes may have affected our results^19^. Further research to investigate the relationship between gene mutation and strabismus in patients with RP is necessary. Finally, the GP area was measured in only 33 patients. Further studies with a larger sample size are necessary to verify our findings.

In conclusion, our findings suggest that visual function is negatively associated with ocular deviation in patients with RP. The risk of sensory strabismus is relatively high among patients with a binocular visual field <40 cm^2^ on GP images. These findings warrant measures for care regarding ocular alignment in patients with RP and severe vision loss.

## Methods

This single-centre, cross-sectional study was approved by the ethics committee of Kyoto University Graduate School of Medicine (Kyoto, Japan). All study protocols adhered to the tenets of the Declaration of Helsinki, and all study participants provided written informed consent.

### Subjects

**Figure 2:**
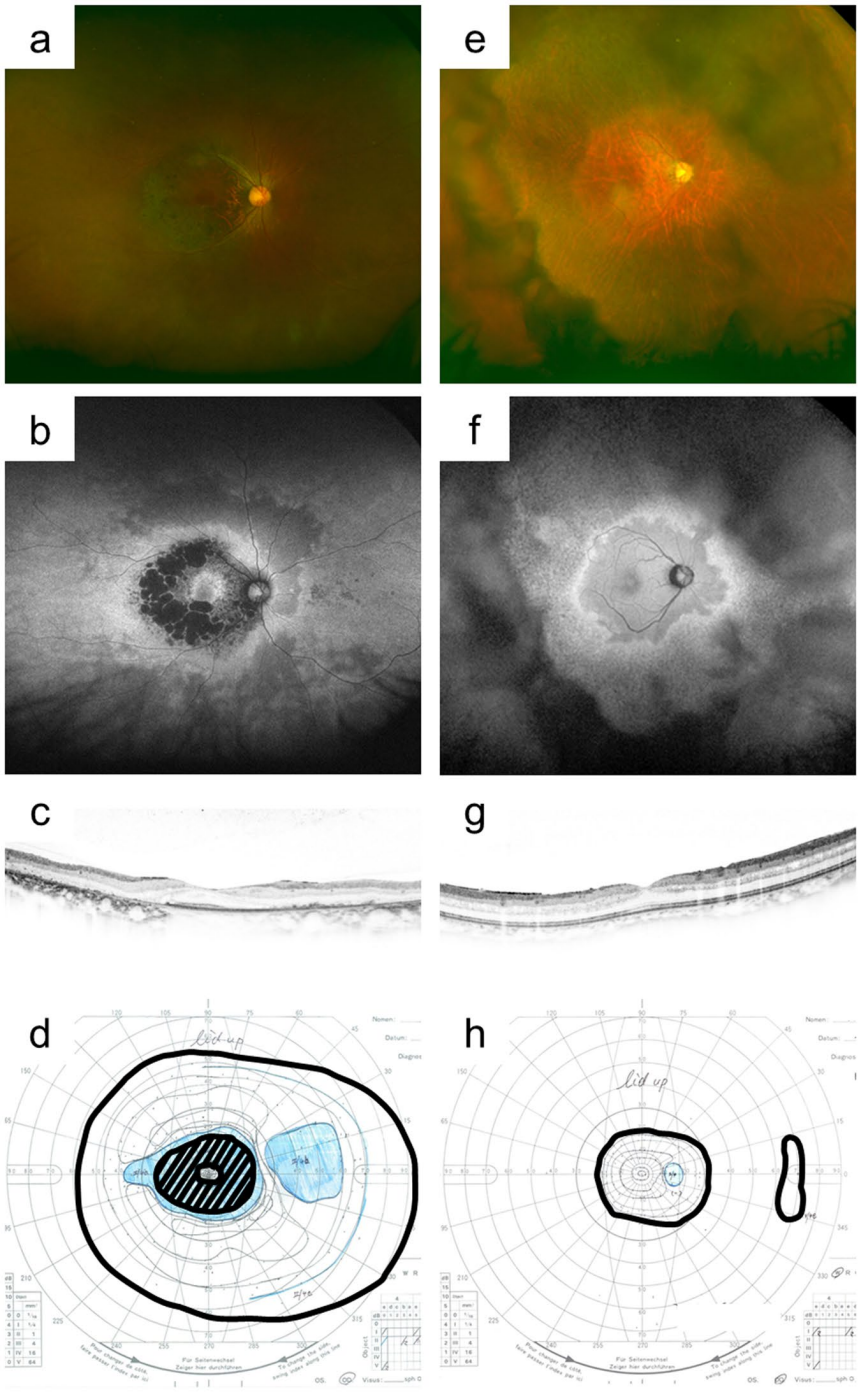
Representative colour and autofluorescence fundus photographs and optical coherence tomography (OCT) and Goldmann perimetry (GP) images for patients with retinitis pigmentosa (RP) and orthophoria or large-angle strabismus. (**a**–**d**) Representative right-eye images from a 40-year-old man with RP who exhibited a mean deviation (MD) value of −33.4 dB, residual GP area of 128.2 cm^2^, and no horizontal or vertical deviation at far distance. (**a**,**b**) Colour and autofluorescence fundus photographs. (**c**) Spectral-domain OCT image. (**d**) GP image. (**e**,**f**) Representative right-eye images from a 52-year-old man with severe RP who exhibited an MD value of −3.4 dB, residual GP area of 28.1 cm^2^, horizontal exodeviation of 15.4°, and vertical deviation of 1.7° at far distance. (**e**,**f**) Clour and autofluorescence fundus photography. (**g**) OCT image. (**h**) GP image. Ocular alignment is stable despite the relatively low MD value and high residual GP area. The bold lines on GP images (**d**,**h**) represent V/4e isopters.

We recruited consecutive patients with RP and no history of strabismus surgery who visited the Department of Ophthalmology and Visual Science at Kyoto University Graduate School of Medicine between June 2015 and November 2017. All patients underwent comprehensive ophthalmological examinations, including autorefractometry; measurement of best-corrected VA, using a decimal VA chart (Landolt chart); indirect ophthalmoscopy; slit-lamp biomicroscopy; colour fundus photography and fundus autofluorescence imaging, using an Optos device (Optos PLC; Dunfermline, UK; Fig. 2); spectral-domain OCT (Spectralis HRA + OCT; Heidelberg Engineering, Heidelberg, Germany); and assessment of MD value with a Humphrey field analyser (Carl Zeiss Meditec, Inc., Dublin, CA), using the 10-2 Swedish Interactive Threshold Algorithm standard program. RP was diagnosed by retinal specialists. From March 2016, the axial length was additionally measured using an IOL Master device (Carl Zeiss Meditec). Ocular deviation was measured using PACT at far (5 m) and near (0.3 m) distances (HDF, HDN, VDF, and VDN) after confirmation of the binocularity status (phoria, phoria–tropia, or tropia) using single cover testing. We considered horizontal and vertical deviation separately because it is unclear whether sensory strabismus mainly induces horizontal deviation. For patients who could not gaze at a fixation target, ocular deviation was measured using the Krimsky test at near distance. The patients were assigned to two groups on the basis of HDN; those with ocular deviation ≥10° and those with ocular deviation <10°; the latter were assigned to large- and small-angle groups, and the characteristics of the two groups were determined.

### Subanalysis of peripheral visual field using Goldmann perimetry

**Figure 3:**
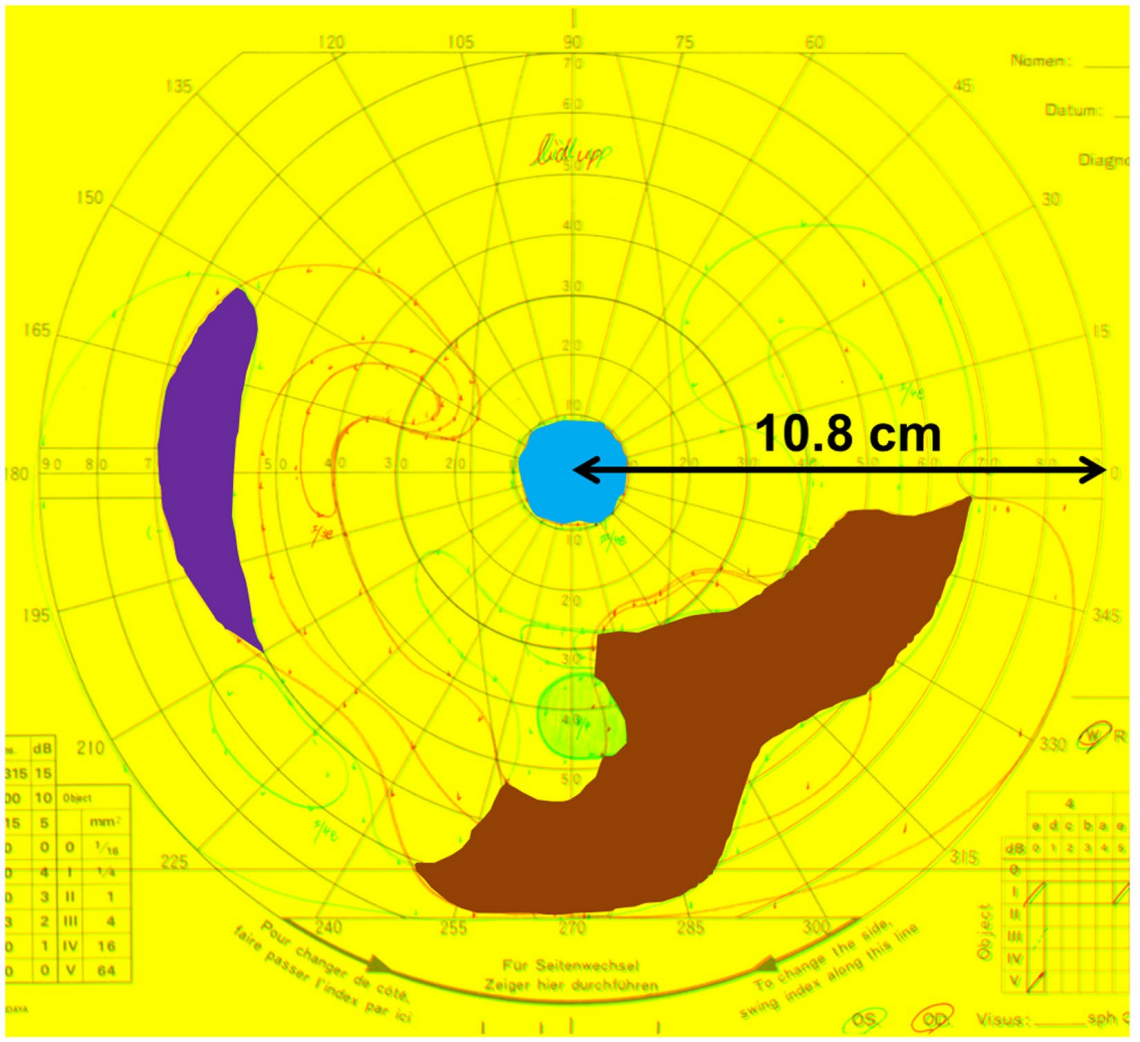
Method for measuring the binocular visual field on merged Goldmann perimetry (GP) images for patients with retinitis pigmentosa. GP findings for both eyes were scanned and merged. The binocular visual field was measured and summated (right eye, red line; left eye, green line). The radius of the central 90° line (between the two arrow heads) was first set at 10.8 cm and measured on a standard recording paper. On this complex image, the residual GP area is 49.38 cm^2^ (blue, purple, and brown areas: 3.62, 8.71, and 37.05 cm^2^, respectively).

GP was performed when clinicians judged that it was clinically required. The binocular visual field area was measured after merging the GP images for both eyes using an open-source software (ImageJ; National Institutes of Health, Bethesda, MD; Fig. 3). The radius of the central 90° line was determined as 10.8 cm on a standard recording paper, and the measurements on the digital images were accordingly calibrated^20^. The isopter of the V/4e white test light was traced, and the sum of the residual binocular visual field areas for the right and left eyes was determined as the residual GP area. The residual GP area was used for the overall analysis and subanalysis for investigating the association between wide visual field and ocular deviation in patients who underwent GP, had an adequate VA for undergoing PACT at far distance, and did not show an interocular difference in VA (logMAR) ≥0.5. Furthermore, we assigned patients included in the subanalysis into phoria (including phoriatropia) and tropia groups and analysed the difference between groups.

### Statistical analysis

Data are presented as means ± standard deviations where applicable. All VA values were converted to logMAR units for statistical analysis. Ocular deviation measured in prism dioptres was converted to degrees (°) and analysed using absolute values, regardless of the strabismus type. The average logMAR and MD values for the right and left eyes and the interocular difference in these values were used as statistical parameters. In accordance with a previous report^21^, patients with a VA of count fingers, hand motion, light perception, and no light perception were arbitrarily assigned logMAR values of 2.6, 2.7, 2.8, and 2.9, respectively. For patients who could not undergo visual field examination with the Humphrey field analyser, the minimum MD value according to age was used in the analysis. Phoria, phoria–tropia, and tropia were assigned values of 1, 2, and 3, respectively, for analysis. Comparative analyses were performed using the *t*-test, chi-square test, and chi-square trend test where applicable. Correlation analyses were performed using Spearman’s rank correlation coefficients. All statistical analyses were performed using SPSS software (version 21, IBM, NY). A *P*-value of < 0.05 was considered statistically significant.
